# Ciprofloxacin and Azithromycin Antibiotics Interactions with Bilayer Ionic Surfactants: A Molecular Dynamics Study

**DOI:** 10.1021/acsomega.4c04673

**Published:** 2024-07-17

**Authors:** Sriprasad Acharya, Jitendra Carpenter, Muddu Madakyaru, Poulumi Dey, Anoop Kishore Vatti, Tamal Banerjee

**Affiliations:** † Department of Chemical Engineering, Manipal Institute of Technology (MIT), Manipal Academy of Higher Education (MAHE), Manipal, Karnataka 576104, India; ‡ Department of Materials Science and Engineering, Faculty of Mechanical Engineering (ME), Delft University of Technology, 2628 CD Delft, The Netherlands; § Department of Chemical Engineering, Indian Institute of Technology Guwahati, Guwahati, Assam 781039, India

## Abstract

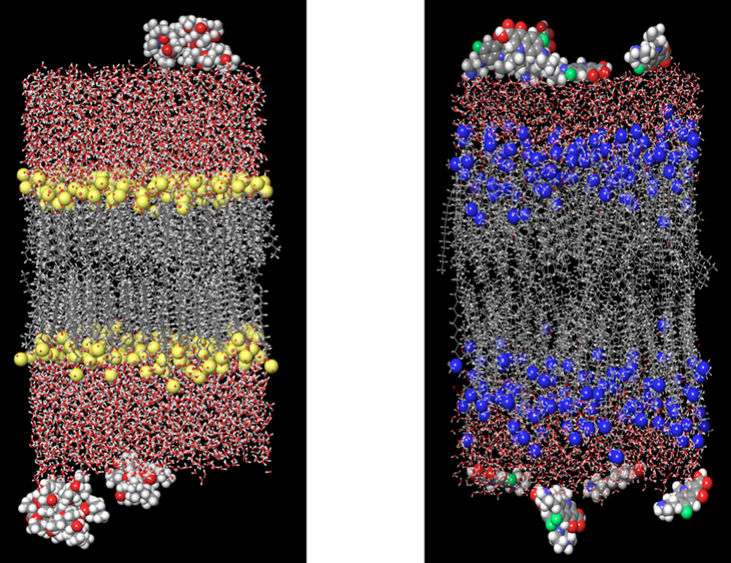

The introduction of pharmaceuticals into aquatic ecosystems can lead to the generation of antibiotic-resistant bacteria. This paper employed molecular dynamics simulations to examine the interactions between cationic/anionic surfactants and two antibiotics or drugs, namely, ciprofloxacin and azithromycin. The analysis focused on many factors to elucidate the mechanism by which the surfactant bilayer molecular structure affects the selected antibiotics. These factors include the tilt angle, rotational angle of the surfactants, electrostatic potential, and charge density along the bilayers. Our molecular-level investigation of the adsorption mechanisms of hydrophobic (azithromycin) and hydrophilic (ciprofloxacin) drugs on the cationic/anionic surfactant bilayer offers a crucial understanding for comprehending the optimal selection of surfactants for effectively separating antibiotics.

## 1 Introduction

Antibiotics have revolutionized the field of medicine by providing effective treatment for bacterial infections. However, the overuse and improper disposal of antibiotics have made them find their way into our water resources, thereby causing detrimental effects on aquatic ecosystems and human health.^1^ Furthermore, the discharge of antibiotics into the aquatic environment, resulting from the processes of the pharmaceutical industry and sewage waste, has instigated a worldwide surge in antimicrobial resistance,^2−4^ making it more difficult to treat infections in both humans and animals. Additionally, antibiotics can disrupt the balance of microorganisms in aquatic environments, influencing the overall health of these ecosystems.^5,6^ It is thus important to monitor and find possible ways to isolate these drugs from water sources using secondary techniques such as foam fractionation.^7^ Numerous works have been performed on removing or minimizing the antibiotic concentrations in water with the help of various techniques such as adsorption,^8,9^ advanced oxidation processes,^10,11^ membrane filtration,^12,13^ biological treatment methods,^14,15^ and using various surfactants.^16−19^

Surfactants form an essential component in the drug industry for their ability to improve the therapeutic concentration of drugs by enhancing their bioavailability.^20^ These amphipathic molecules form micelles that reduce surface tension in various systems, enhancing solubility, dissolution profile, permeation, and stability. Surfactants could be anionic, cationic, nonionic, or amphoteric, each with their own unique properties and applications.^21^ They are commonly used in soaps, detergents, and industrial processes, leading to excessive concentrations in water bodies that pose environmental and toxicology issues due to their persistent nature.^22^ Anionic surfactants, such as sodium dodecyl sulfate (SDS), are commonly used in cleaning products due to their ability to remove dirt and grease. Cationic surfactants, such as cetyltrimethylammonium bromide (CTAB), are often used in fabric softeners and hair conditioners for their ability to reduce static electricity. Nonionic surfactants, such as poly(ethylene glycol), are gentle on the skin and are commonly found in personal care products. Amphoteric surfactants, like cocamidopropyl betaine, have both positive and negative charges, making them versatile in a variety of applications.^23^ The combined presence of surfactants and antibiotics in water bodies has increased manifold in recent decades due to their broad usage. Their synergistic coaction can increase the bioavailability of antibiotics, leading to higher levels of these drugs in aquatic systems. This can further exacerbate the development of antibiotic-resistant bacteria and disrupt the natural equilibrium of microbial communities. Therefore, understanding the interactions between surfactants and antibiotics is essential in developing effective strategies to mitigate their impact on water quality and ecosystem health, thereby protecting the environment and preventing potential health risks for both aquatic organisms and humans.

Numerous experimental investigations have been performed to probe the interactions between antibiotics and surfactants. Rahman et al.^24^ experimentally investigated the interaction of ceftriaxone sodium trihydrate (CFT) drug with ionic and nonionic surfactants. The micellization of tetradecyltrimethylammonium bromide (TTAB) with CFT was studied based on conductivity measurements, whereas Triton X-100 (TX-100) and Tween 80 with CFT were based on cloud point measurement. The conductivity was measured by varying the concentration of the surfactants. A breakdown in the linearly increased conductivity is observed after a certain concentration, indicating micelles formation. Based on the conductivity results, on incorporating the CFT drug (at a concentration of 0.5 mmol/kg), the critical micellar concentrations (CMC) of the TTAB system were increased to 4.56 from 3.7 mmol/kg of pure TTAB system at 310 K. The nature of hydration (i.e., hydrophilic or hydrophobic) was hypothesized based on increased or decreased CMC values at different temperatures. Similarly, the interaction of CFT with TX-100 was explored based on the cloud point conditions (*T*_CP_-temperature at which cloud point is observed) at different surfactant and CFT concentrations. The *T*_CP_ was found in the range of 332.43–339.75 K at the concentrations range of 1–10% (w/w) in the aqueous phase. A marginal increase in *T*_CP_ of TX100 in the presence of CFT indicated the formation of micelle aggregation carrying the drug. The interaction of CFT with Tween 80 was investigated using the UV spectrophotometric method. It was found that the maximum wavelength of pure CFT increases in the presence of Tween 80, indicating the existence of interaction between them. Similar work^25^ was also conducted by the same group on the interaction of the same drug (CFT) with the CTAB. In this study, the intermolecular interaction was investigated based on the micellization of surfactant in the aqueous phase in the presence of the drug at different CTAB concentrations. In another analogous work by Ahsan et al.,^26^ the interaction between moxifloxacin hydrochloride (MFH) with SDS was studied. The interaction was elucidated by measuring the CMC based on the conductivity at different surfactant concentrations and temperatures in the aqueous phase. It was observed that on increasing the surfactant concentration, the conductivity was initially increased and then decreased due to the formation of the surfactant assembly. The conductivity results showed that with the incorporation of MFH, the CMC of SDS was found to be almost 1.5 times more than that of pure SDS in the aqueous phase. Moreover, the CMC was also measured as a function of temperature in the range of 298–318 K for the SDS/MFH system, and it was found that the CMC decreases with an increase in temperature due to the reduction of the hydration effects. Pathania et al.^27^ experimentally investigated the interaction between cefepime drug and CTAB and DTAB surfactants using densimetry and sound speedometry. The CMC of surfactants was determined based on the speed of sound data at different temperatures. The encapsulation of drugs in surfactants and their intermolecular interaction were evident in the Fourier-transform infrared spectroscopy (FTIR) and nuclear magnetic resonance spectroscopy (NMR) results.^27^ Rub et al.^17^ reported the micellization behavior of amitriptyline hydrochloride (AMT) and a surfactant triton X-45 (TX-45). The CMC was measured based on the effect on surface tension at different concentrations of surfactant in drug-surfactant mixtures. The amount of surfactant added at which no further reduction in surface tension was observed was considered as a CMC of the system. It was observed that the CMC of the mixed drug-surfactant system in the aqueous phase was found to be lesser than the pure surfactant system.^17^ On increasing the concentration of the drug, the CMC was significantly reduced, which indicated the micellization of the mixed process via attractive interactions. The micellization of surfactant in the system was also confirmed based on the obtained activity coefficients. The surfactant activity coefficients were less than the AMT activity coefficients, indicating the presence of a high amount of surfactant in the mixed micellar system. Their findings suggested that the TX-45 surfactant can facilitate the delivery of the AMT drug and enhance its bioavailability. Similar observations were also made in the study reported by Kumar et al.^28^ for a system of ibuprofen with surfactants, hexadecyltrimethylammonium bromide (HTAB), and a gemini surfactant. The interaction of ibuprofen with both surfactants was investigated and compared based on the surface and thermodynamic properties of the systems. Based on the parametric investigations, it was found that the hydrophobicity of the ibuprofen-gemini surfactant mixture was more than the ibuprofen-HTAB mixture, indicating the favorable micellization and more interaction of the ibuprofen-gemini surfactant.

Several experimental and theoretical investigations have been performed employing surfactant bilayers as well.^29^ Zhang et al.^30^ performed molecular dynamics (MD) simulations of a bilayer consisting of SDS molecules in conjunction with a solid surface. Their work proposed a potential mechanism for the creation of curved SDS bilayers. The aggregates undergo a transition from curved bilayers to planar bilayers, to perforated bilayers, and finally to micelles as the cross-sectional area increases. Tang et al.^31^ performed MD simulations of preassembled SDS micelles utilizing several force fields, namely, GROMOS, CHARMM36, OPLS-AA, and OPLS-UA. These simulations were performed for varying aggregation numbers and box sizes. The variation in force fields was found to have a minimal impact on the overall micelle structure of small aggregates with sizes of 60 or 100. However, for micelles with an aggregation number of 300 or greater, bicelle structures with organized tails were observed instead of the more realistic rodlike micelles with disordered tails.

Reverse micelles have garnered significant interest in recent decades as an innovative approach to the separation and purification of antibiotics. This is due to their ability to create a unique microenvironment within the organic medium.^32^ Zhang et al.^33^ conducted a study on the separation of bovine serum albumin (BSA) using a cationic surfactant, i.e., CTAB. At a pH of 7.4, which is higher than the isoelectric point of BSA, a maximum recovery ratio of 80.5% was achieved. Xu et al.^34^ conducted a study on the adsorption of BSA in a foam fractionation process utilizing cationic, anionic, and nonionic surfactants. Their research showed that in the presence of both cationic and anionic surfactants, BSA migrated to the gas–liquid interface owing to a strong attraction.

Boukhelkhal et al.^32^ worked on the experimental investigations to remove amoxicillin from aqueous solution using the anionic surfactant, i.e., SDS. The study unravels the optimal adsorption kinetic parameters, such as contact time, pH, temperature, and initial concentration of SDS. A removal efficiency of 87.7% of amoxicillin was observed by the authors. Huang et al.^35^ developed a two-stage batch foam fractionator to recover creatine from wastewater using the SDS. The authors observed optimum operating conditions with an enrichment ratio of 3:1, leading to a recovery percentage of 70.6. Ghosh et al.^7^ worked on the adsorption of fluoroquinolone antibiotics by employing a semibatch foam fractionation process. In their work, two surfactants were considered, i.e., cationic CTAB and anionic SDS. It was observed that ciprofloxacin partitions to the gas–liquid interface more readily in the presence of SDS compared to CTAB. The authors found higher removal efficiency with SDS (96.3%) in comparison to CTAB (52%) for the ciprofloxacin.

To briefly summarize the above discussion, the potential synergistic or antagonistic effects between surfactants and antibiotics must be thoroughly investigated to predict the effects caused by the interaction of these components in our water bodies. Understanding how different surfactants interact with specific antibiotics can lead to the development of more effective methods to isolate antibiotics efficiently. To handle this issue, we probed the SDS/CTAB bilayer interactions with the ciprofloxacin/azithromycin drugs. We have thoroughly analyzed the tilt angle, rotational angle of the surfactants, hydrogen bonds between drug and surfactant, density profile of drugs, electrostatic potential, and charge density along the bilayers. Our results explain the atomistic interaction mechanisms between the surfactants and antibiotics, which is lacking in the existing literature.

## 2 Computational Details

Within this study, the DESMOND^36^ simulation engine was used within Schrödinger simulation software.^37^ The bonded and nonbonded interactions were described using the Optimized Parameters for Liquid Simulations (OPLS4)^38^ force fields. Temperature control was accomplished using a Nose-Hoover Chain thermostat. Pressure control was applied using the Martyna-Tobias-Klein semi-isotropically at 300 K and 1.0 atm, and the relaxation times for the thermostat and barostat were 1 and 2.0 ps, respectively. A time step of 2 fs was used for the OPLS4 force field simulations. We performed 100 ps of Brownian minimization followed by 0.1 ns of NVT MD run (*T* = 10 K and time step = 1 fs) to slowly introduce the dynamics. Later, 20 ns of the NPγT run was performed (γ is surface tension) at 300 K and 1 atm. We have considered γ = 33.0 mN m^–1^^39,40^ for SDS and γ = 36.5 mN m^–1^^41^ for the CTAB bilayer.

**Figure 1:**
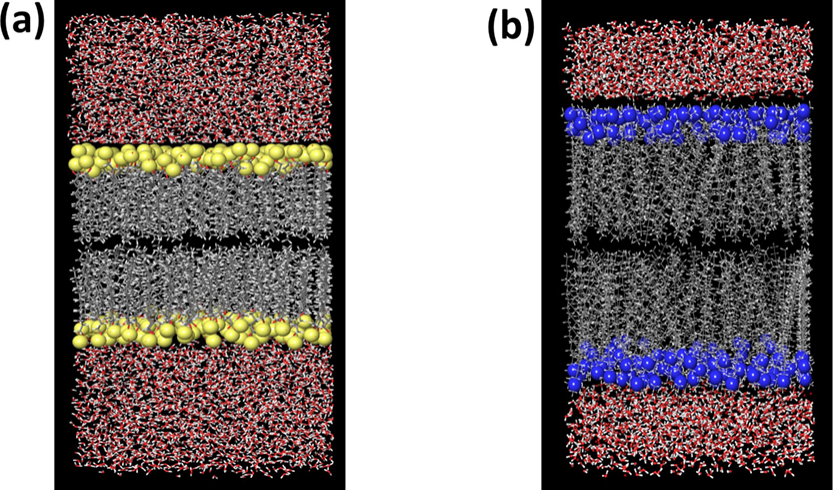
(a) shows the snapshot of the MD run of SDS bilayer in an aqueous environment (b) shows the snapshot of the MD run of CTAB bilayer in an aqueous environment. Sulfur of SDS surfactant is shown in yellow color visualized in CPK model. Nitrogen atoms of CTAB surfactants are shown in blue color visualized in the CPK model. Water molecules are visualized in the ball and stick model. Gray color denotes the hydrophobic tail of surfactant.

**Figure 2:**
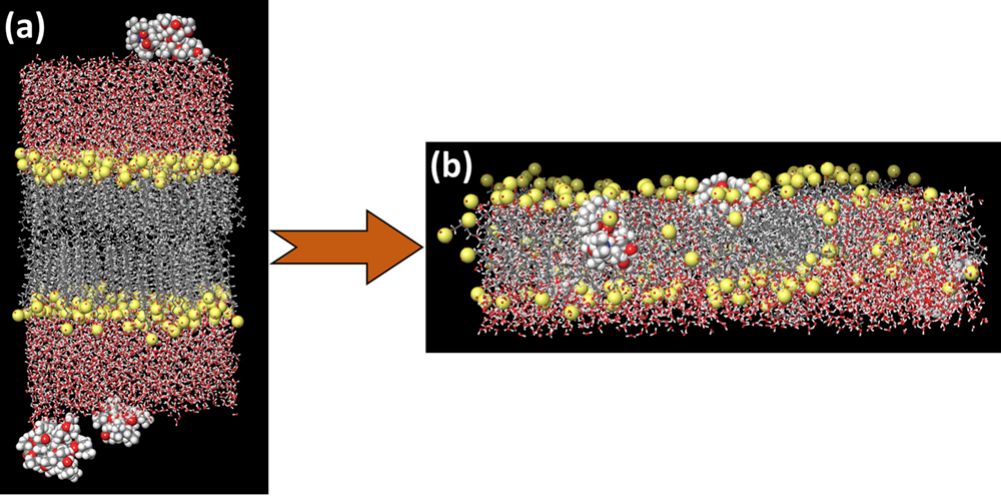
(a) Snapshot of the MD run of SDS Bilayer in an aqueous environment along with ciprofloxacin before the NPT run. (b) Snapshot of the MD run of SDS bilayer in an aqueous environment along with ciprofloxacin after the NPT run. Sulfur of the SDS surfactant is shown in yellow color visualized in the CPK model. Ciprofloxacin is visualized in the CPK model. Water molecules are visualized in the ball and stick model.

**Figure 3:**
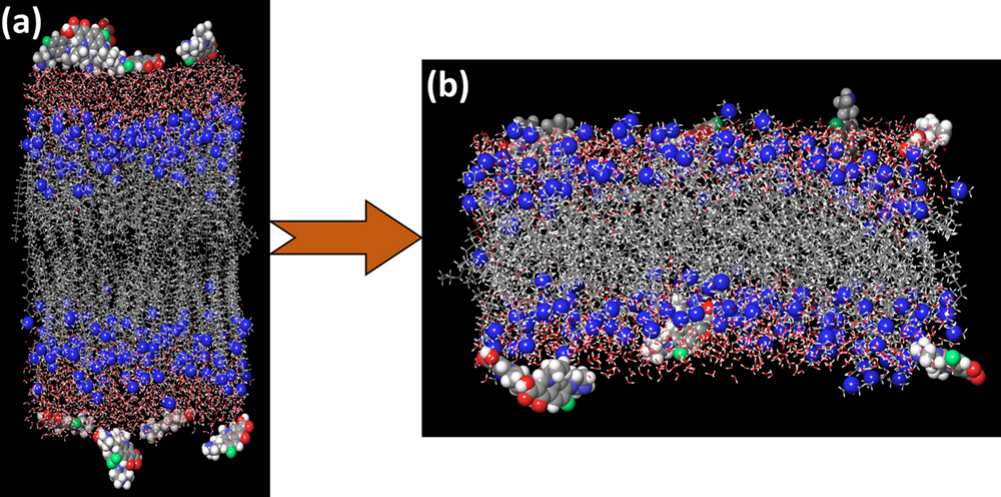
(a) Snapshot of the MD run of CTAB Bilayer in an aqueous environment along with azithromycin before the NPT run. (b) Snapshot of the MD run of CTAB Bilayer in an aqueous environment along with azithromycin after the NPT run. Nitrogen atom of the CTAB surfactant is shown in blue color visualized in CPK model. Azithromycin is visualized in the CPK model. Water molecules are visualized in ball and stick model.

Surfactant molecules were placed in a way that avoids atomic overlap, ensuring an initial configuration as shown in Figure 1. Atomic overlap was defined based on the van der Waals radii of the atoms, a theoretical hard sphere that represents the distance of closest approach between two atoms. The packing efficiency factor was used to uniformly scale the van der Waals radii of the atoms. This quantity directly impacts the density of the output structure. We used the packing efficiency factor of 0.6. We considered two drugs, mainly hydrophilic and hydrophobic drugs, i.e., ciprofloxacin and azithromycin. 4–8 wt % of the drug in aqueous solution was considered. Ciprofloxacin and azithromycin drugs were added to the surfactant bilayer, as shown in Figures 2a and 3a. Table 1 lists the number of molecules and box sizes considered for each simulation performed in this study.

**Table 1:** Number of Molecules and Box Sizes Considered for Each Simulation Run Performed in This Study

no. of surfactants molecules	no. of ciprofloxacin	no. of azithromycin	no. of water molecules	box size before NPT run (Å^3^)	box size after NPT run (Å^3^)
250 (SDS)			4962	52.05 × 52.16 × 102.00	95.54 × 95.74 × 27.95
250 (SDS)	10		4962	52.05 × 52.16 × 127.15	97.16 × 97.36 × 27.67
250 (SDS)		5	4962	52.05 × 52.16 × 135.14	98.33 × 98.53 × 26.99
228 (CTAB)			2394	52.08 × 52.17 × 82.00	85.75 × 85.90 × 28.85
228 (CTAB)	10		2394	52.08 × 52.17 × 107.15	87.48 × 87.64 × 28.33
228 (CTAB)		5	2394	52.08 × 52.17 × 116.18	86.37 × 86.52 × 29.27

## 3 Results and Discussion

### 3.1 Surfactant Tilt and Rotational Angles

**Figure 4:**
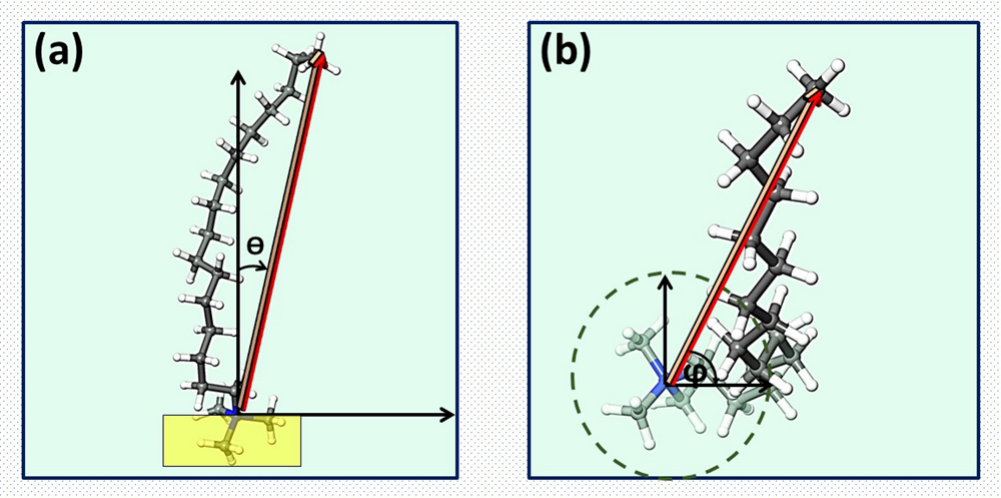
Schematic representation of the calculated (a) Surfactant tilt angle and (b) Surfactant rotation angle. θ represents tilt angle and φ represents rotational angle.

The distribution of the tilt and rotational angles provides information about the alignment or randomness of surfactants. The surfactant tilt angle and rotational angle are shown in Figure 4a,b. The tilt angle (θ) of a surfactant is the angle between the normal vector of a plane representing the surface and the vector extending from the surface to the hydrophobic end of the surfactant, indicating the orientation of the surfactant. The surface plane can be defined using a variety of approaches. In this work, the best fit for specific atoms is considered as shown in the yellow highlighted region in Figure 4 in the tilt angle schematic. In order to determine the rotational angle (φ) of a surfactant, the orientation vector is projected onto the surface plane. The angle between the projected vector and the *x*-axis represents the rotational angle of the surfactant.

**Figure 5:**
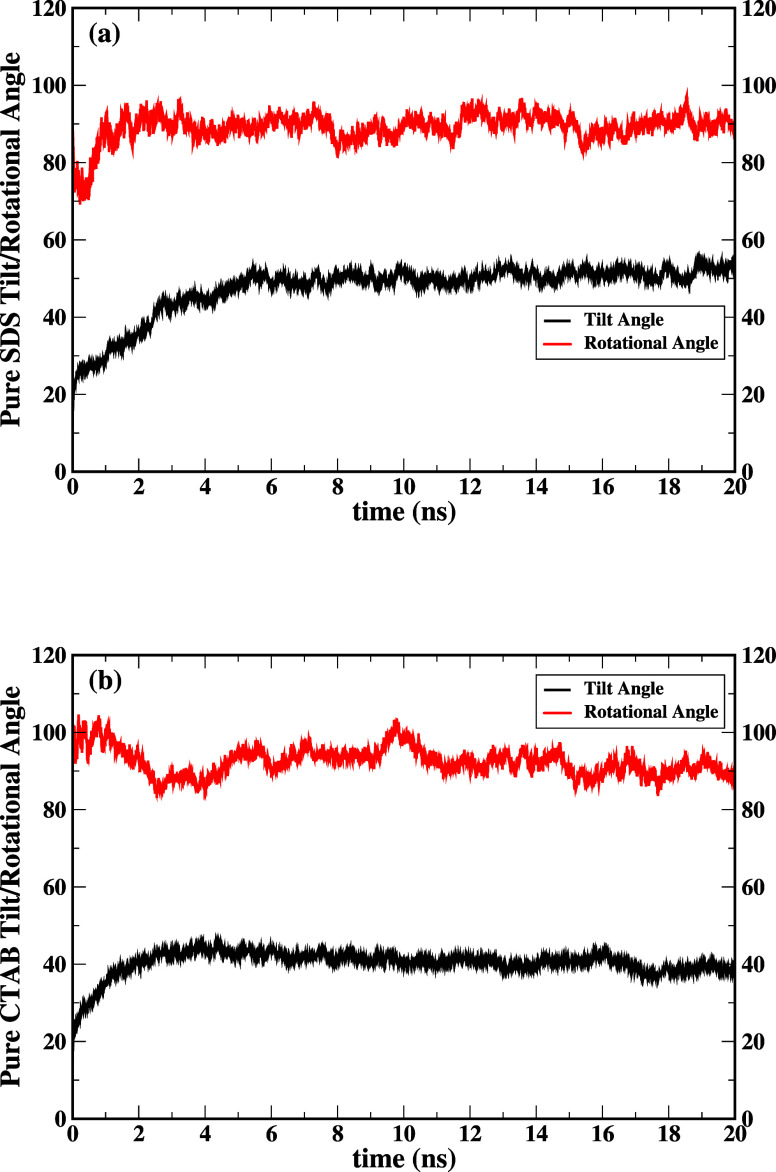
(a) Surfactant tilt and rotational angles of pure SDS versus time in aqueous solution. (b) Surfactant tilt and rotational angles of pure CTAB versus time in aqueous solution. Black line represents the tilt angle and the red line represents the rotational angle.

The time series surfactant tilt angle and rotational angle of the pure SDS and CTAB are shown in Figure 5a,b. Frequency distribution versus tilt/rotational angle is analyzed over 20 ns simulation time. The time-averaged tilt angle and rotational angle are listed in Table 2. The surfactant tilt angle of pure SDS surfactant in aqueous solution is compared with the presence of SDS and antibiotics in water. The tilt angle of the pure cationic SDS is found to be 47.416°, whereas a slight change is observed in the presence of both drugs as presented in Table 2. On the other hand, the tilt angle of the anionic CTAB is found to be independent of both the selected drugs, i.e., ca. 41°. The ciprofloxacin is a hydrophilic drug and it interacts with the hydrophilic head of the surfactant. This is the reason the tilt angle changes are minimum in the absence and presence of ciprofloxacin. It is interesting to note that the pronounced changes in the rotational angle are visible in the presence of both the drugs. We observed an interaction between the azithromycin hydrophobic drug and the hydrophobic tail of the SDS surfactant, due to which the rotational angle is changed from 89.114 to 90.353° corresponding to the change from pure SDS to SDS+Azi, whereas in the presence of ciprofloxacin, the rotational angle is found to be 90.463°. Furthermore, we noticed a decrease in rotational angle for the CTAB surfactant, where the rotational angle is changed from 92.443 to 87.453° corresponding to the change from pure CTAB to CTAB+Azi, whereas the rotational angle is found to be 92.885° in the presence of ciprofloxacin.

**Table 2:** Calculated Surfactant Tilt and Rotational Angle for the Considered Systems along with the Standard Deviation of Time Series for Both the Tilt Angle and Rotational Angle Are Shown in the Table

system	tilt angle (°)	tilt angle standard deviation (°)	rotational angle (°)	rotational angle standard deviation (°)
Pure SDS	47.416	6.73	89.114	3.56
SDS+Cipro	45.771	6.98	90.463	3.36
SDS+Azi	46.244	6.22	90.353	3.30
Pure CTAB	40.300	3.19	92.443	3.50
CTAB+Cipro	40.966	4.08	92.885	3.25
CTAB+Azi	41.901	7.74	87.453	4.65

### 3.2 Electrostatic Potential and Charge Density

Ciprofloxacin being zwitterionic, it is important to probe the electrostatic potential. By utilizing the charge density, one can examine the configuration of charges within the system. Electrostatic potential measures the quantity of work required to transfer a unit charge from one reference point to another in the electric field generated by the surrounding environment. It is calculated directly from the charge density. In bilayer systems, the electrostatic potential ϕ is determined by calculating its value at different radial distances from a reference point which serves as the center of the trajectory. The reference point (*r* = 0) is established as the central location of the bilayer, and the electrostatic potential is then determined by integrating the electric field radially.
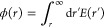
1

The radial electric field *E*(*r*) is defined as follows:
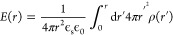
2

The symbol ϵ_s_ represents the relative permittivity of the solvent, ϵ_0_ represents the vacuum permittivity, and ρ represents the charge density. The calculation of the charge density relies on the formal charge assigned to each individual atom. Atoms that have a formal charge of 0 are ignored. The electrostatic potential is nullified at radial distances far from the center of the bilayer.

For interfaces that are flat and perpendicular to the *z*-direction, the trajectory is segmented into bins of a thickness along the *xy*-plane. The charge density is obtained by averaging the ion distribution from each bin and then integrating it along the *z*-direction to calculate the electrostatic potential. The reference is the *xy*-plane located within the central region of the solvent. The expression for the electrostatic potential ϕ is formulated as
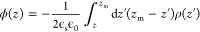
3where *z*_m_ is the *z* value of the reference plane. The electrostatic potential is defined as zero in the reference plane.

**Figure 6:**
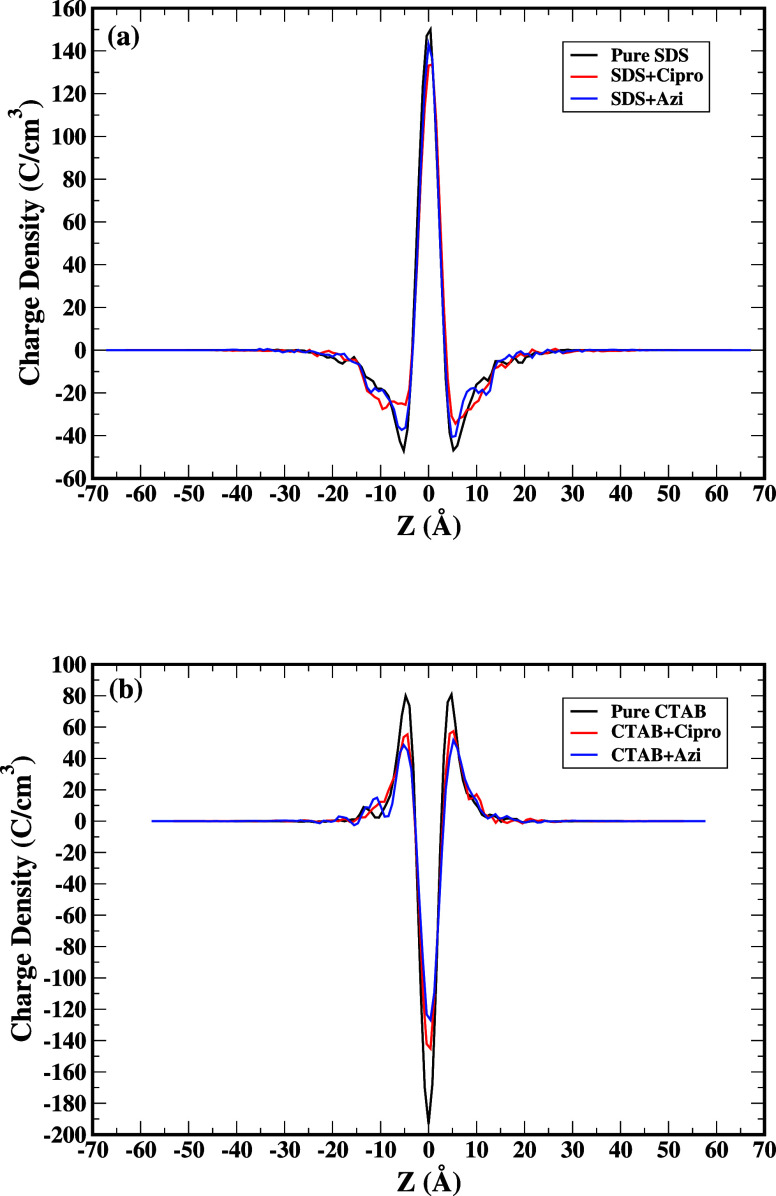
Charge density profile across the bilayer surfactant as a function of *z*-coordinate, which measures the distance from the midplane of the bilayer along the normal direction. (a) pure SDS, SDS+Cipro and SDS+Azi (b) pure CTAB, CTAB+Cipro and CTAB+Azi. Black line represents charge density of pure surfactant in aqueous solution, red line represents charge density of surfactant + ciprofloxacin in aqueous solution, and blue line represents charge density of surfactant + azithromycin in aqueous solution.

Figure 6a displays the charge density as a function of the distance from the center of the solvent in the *z* dimension, which is perpendicular to the SDS bilayer. The value of *z* = 0 represents the centrality of the bulk solution, determined by the aqueous solvent in the calculations. This central point is occupied by positively charged sodium ions. The profile exhibits approximate symmetry with respect to *z* = 0 due to the symmetric nature of the bilayer geometry. As we move away from *z* = 0 in both directions (See Figure 6a), the average charge density in the region corresponding to the negatively charged sulfate head groups becomes negative. This charge density becomes less negative in the presence of the ciprofloxacin. This is attributed to stronger interactions between the hydrophilic head of the surfactant and ciprofloxacin drug. Continuing deeper inside the bilayer, the charge becomes somewhat positive as a result of a smaller number of sodium ions that enter the surface of the bilayer as shown in Figure 6a. Ultimately, the charge density reaches zero within the uncharged alkane tails. Figure 6b illustrates the relationship between the charge density and distance from the center of the solvent in the *z* dimension, which is perpendicular to the CTAB bilayer. The central point of the bulk solution is occupied by bromide ions, which have a negative charge. As we move away from *z* = 0 in both directions, the average charge density in the region corresponding to the positively charged nitrate head groups becomes positive. As one moves further into the bilayer, the charge becomes increasingly negative, because fewer bromide ions are able to enter the surface of the bilayer.

**Figure 7:**
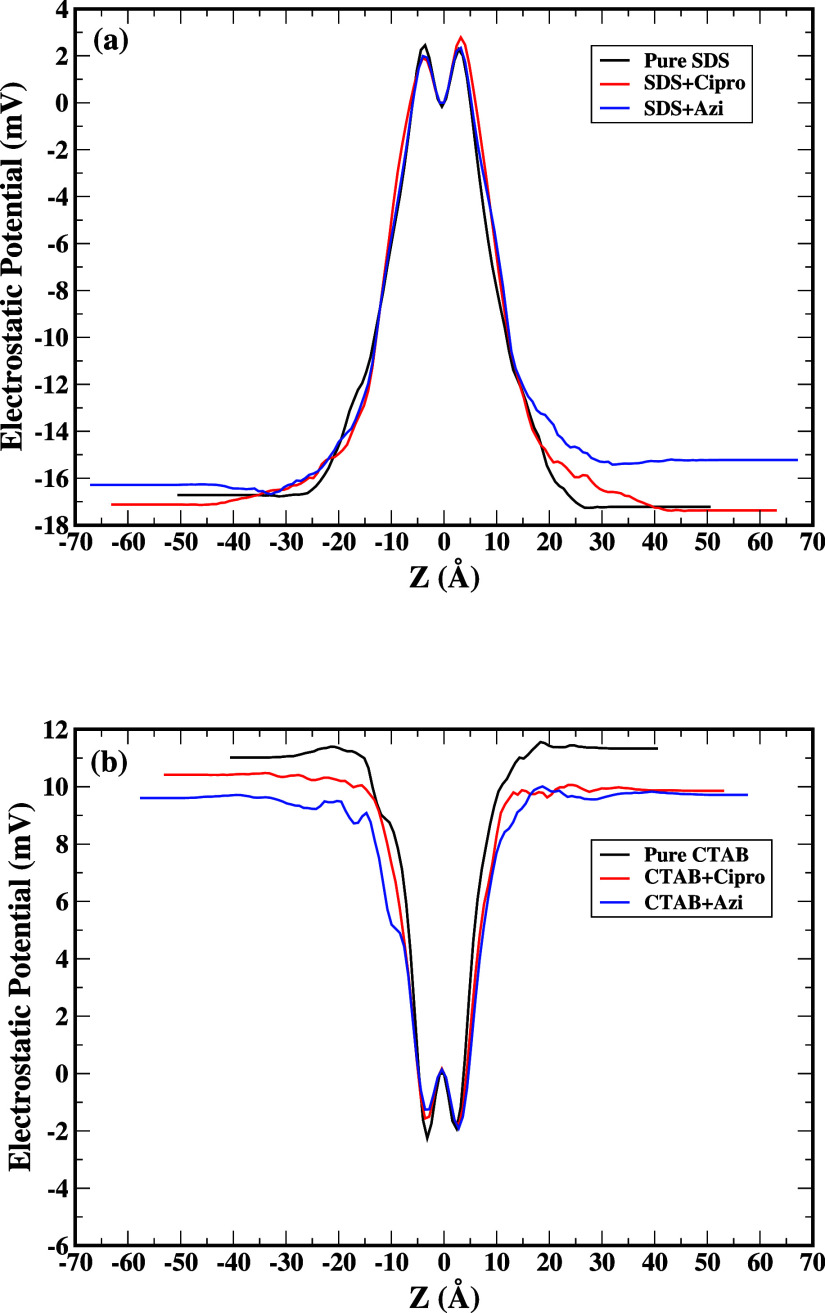
Electrostatic potential profile across the bilayer surfactant as a function of *z*-coordinate, which measures the distance from the midplane of the bilayer along the normal direction. (a) pure SDS, SDS+Cipro and SDS+Azi (b) pure CTAB, CTAB+Cipro and CTAB+Azi. Black line represents electrostatic potential of pure surfactant in aqueous solution, red line represents electrostatic potential of surfactant + ciprofloxacin in aqueous solution, and blue line represents electrostatic potential of surfactant + azithromycin in aqueous solution.

Figure 7 shows the electrostatic potential along the *z* dimension, relative to the reference position at *z* = 0. As mentioned before, the electrostatic potential is the amount of work needed to move a unit charge from a reference point to a second point within an electric field. Therefore, the electrostatic potential at the reference point *z* = 0 is, by definition, 0. Moving within the first 5 Å on either side of the reference point, the electrostatic potential is greater than 0, indicating that work must be performed in order to move a charge from the reference position. The electrostatic potential reaches a minimum in the region corresponding to the negatively charged sulfate groups as shown in Figure 7a. A single positive charge would be more favorable in this position. Finally, the electrostatic potential levels off at a negative value in the region of the alkane tails. Both electrostatic and steric barriers along the way hinder charge diffusion from the highly charged region around the reference point for SDS. Figure 7b shows the electrostatic potential along the *z* dimension, relative to the reference position at *z* = 0. Moving within the first 5 Å on either side of the reference point, the electrostatic potential is less than 0, indicating that less work must be done in order to move a charge from the reference position. The electrostatic potential reaches a maximum in the region corresponding to the positively charged nitrate groups. A single negative charge would be more favorable in this position. Finally, the electrostatic potential levels off at a positive value in the region of the alkane tails for CTAB. Due to the strong interaction of the alkane tails of CTAB with azithromycin, we observe a lesser value than the maximum as shown in the blue line in Figure 7b. The hydrophobic attraction between the large aromatic ring of the quinoline and the extended alkyl chain of the surfactant also plays a role in the binding of the surfactant and drugs. In the SDS-ciprofloxacin system, the carboxyl acid group and carbonyl group created electrostatic repulsion, which shielded the SDS monomers from the quinoline.

### 3.3 H-Bond Interactions

**Figure 8:**
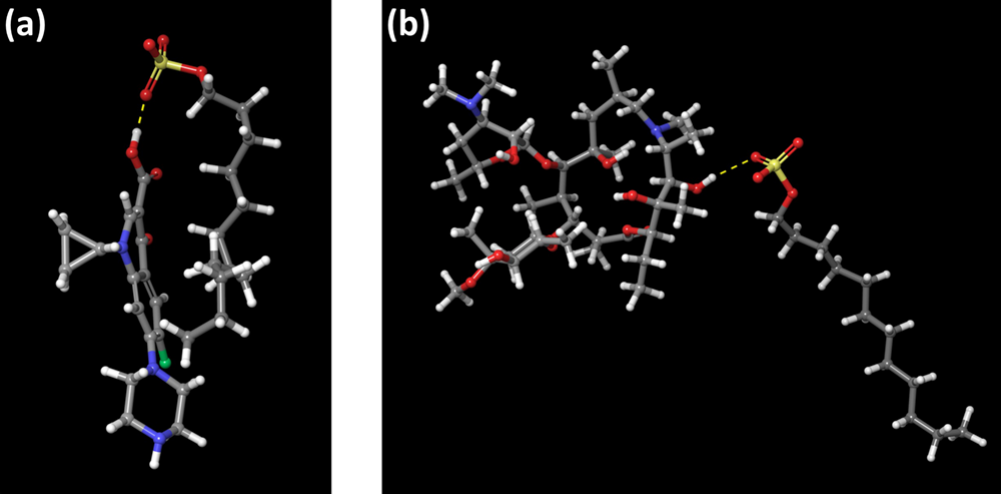
(a) H-bond formation between SDS and ciprofloxacin (b) shows the H-bond formation between SDS and azithromycin. Yellow dashed line represents the hydrogen bond. Drug and SDS surfactant molecules are visualized in a ball and stick model.

**Figure 9:**
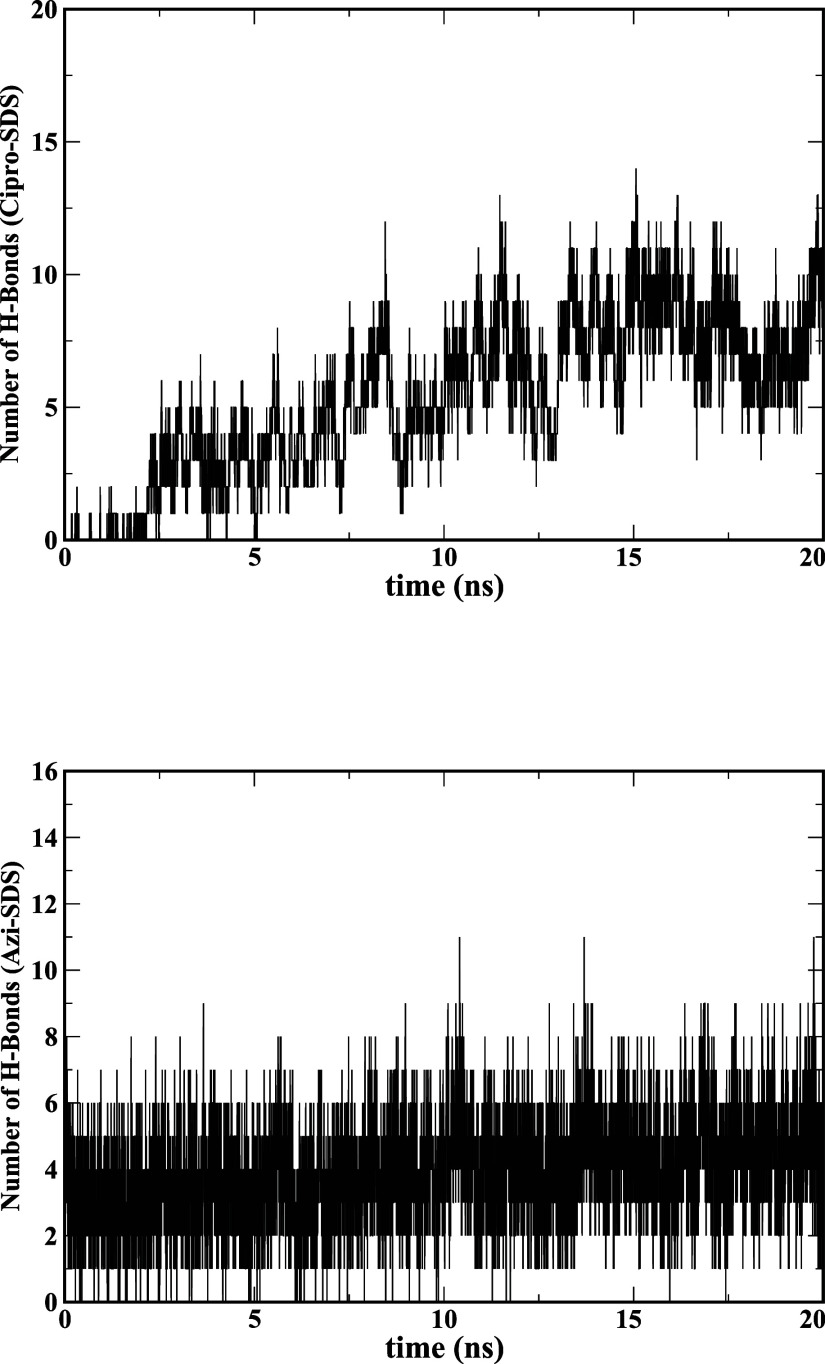
(Top) Number of the H-bonds between SDS and ciprofloxacin pair versus simulation time. (Bottom) Number of the H-bonds between SDS and azithromycin pair versus simulation time.

The H-bond analysis is crucial to understand the nonbonded interactions between surfactants and drugs. The hydrogen bonds can be formed between the hydrogen of the hydroxyl group of the drug and with oxygen of surfactants. The hydrogen bond mechanism between ciprofloxacin-SDS and azithromycin-SDS is shown in Figure 8a, b. Based on the experimental evidence, it was observed that the SDS is more efficient in removing ciprofloxacin than CTAB.^7^ This can be attributed to the formation of the hydrogen bonds between ciprofloxacin and SDS as observed in our study. No hydrogen bonds were observed between CTAB and ciprofloxacin/azithromycin in our study. This can be attributed to the fact that CTAB is known to have neither hydrogen donors nor acceptors.^42^ However, head groups of CTAB can be hydroxylated^43^ to form hydrogen bonds with substrate molecules. The hydrogen bond time series for ciprofloxacin-SDS and azithromycin-SDS are shown in Figure 9 (top) & (bottom). It is evident from the figure that the number of hydrogen bonds between SDS and ciprofloxacin is in the range of 2 to 10, whereas it is in the range of 1 to 8 between SDS and azithromycin.

### 3.4 Drug Density Profiles

Density profiles show the drug distribution within the surfactant bilayer, and it is crucial to understand the localization of the drug at the head/tail of the surfactant. The density profile is determined by dividing the object into layers of a set thickness that are perpendicular to a *z*-axis. In order to determine the density profile at a specific point along the axis (defined as the coordinate at the bottom of the layer), the fraction of the van der Waals volume of each atom that intersects with the layer is multiplied by its atomic mass. The resulting values are then added together for all specified atoms and divided by the volume of the layer.

**Figure 10:**
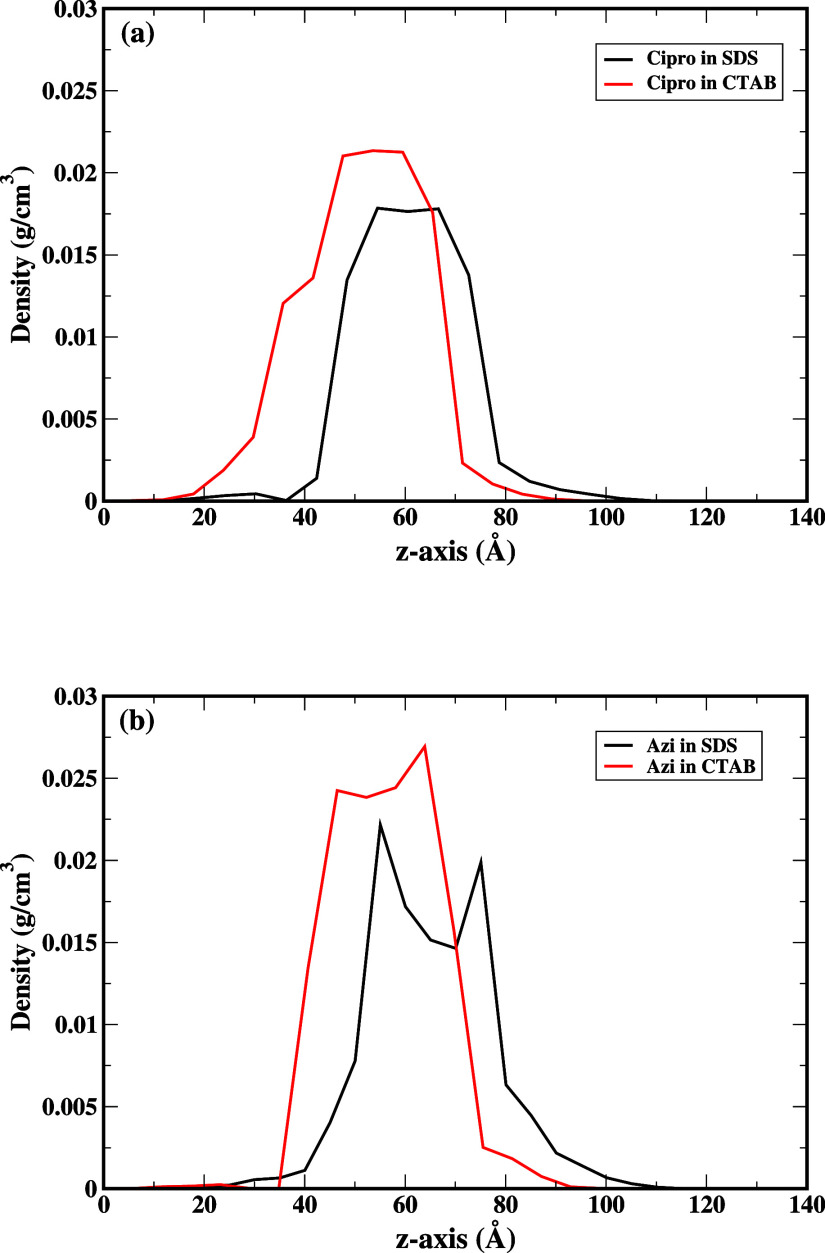
(a) Shows the ciprofloxacin density profile as a function of *z*-axis in SDS and CTAB in an aqueous environment. (b) Shows the azithromycin density profile as a function of *z*-axis in SDS and CTAB in an aqueous environment.

Figure 10a shows the ciprofloxacin density profile in SDS and CTAB bilayers, and Figure 10b shows the azithromycin density profile in SDS and CTAB bilayers. It is evident from Figure 10a that the ciprofloxacin is localized within 40 Å of the SDS bilayer along the *z*-axis, whereas the ciprofloxacin is delocalized within 50 Å in the *z*-axis for the case of the Cipro+CTAB system. This can be attributed to the presence of hydrogen bond interactions between ciprofloxacin and the hydrophilic sulfate group. The delocalization can be attributed to the absence of hydrogen bond interaction between ciprofloxacin and CTAB. Figure 10b shows that the density distribution of the azithromycin in SDS is in the range of 60 Å which can be attributed to the large molecule size of the azithromycin. Azithromycin in CTAB is observed to be localized within 45 Å along the *z*-axis due to the dominant interaction between the CTAB hydrophobic tail and hydrophobic azithromycin.

The synergistic interaction of two amphiphiles is facilitated by various types of molecular interactions. These include electrostatic repulsion between two charged groups, steric repulsion between bulky hydrophilic and hydrophobic groups, ion-dipole interaction between ionic hydrophilic groups, and hydrogen bonding. However, experiments suggest that the electrostatic contacts between the negatively charged surface of SDS micelles and the localized positive charge on the nitrogen atom of moxifloxacin are stronger compared to the interactions between the positively charged surface of dodecyl-trimethylammonium bromide micelles.^44^ It is important to understand the steric effects, as well. We have analyzed how diffusion rates^45^ are kinetically hindered due to the steric effects of the hydrophilic head of the surfactant in the Supporting Information.

The calculated diffusion rates are summarized in Table S1. It can be clearly seen that the mobility of the azithromycin, i.e., 1.425 × 10^–10^ m^2^/s is twice higher than the ciprofloxacin, i.e., 0.786 × 10^–10^ m^2^/s. This can be attributed to the higher number of average H-bonds between SDS-ciprofloxacin than SDS-azithromycin. Enhanced H-bonds interactions along with the steric effect of the hydrophilic head of the surfactant slow down the mobility of the ciprofloxacin. On the other hand, both drug molecules diffuse at almost the same rate along the *z*-axis of the CTAB bilayer, which can be attributed to the steric effects. Overall, the drug diffusion rate is lower in CTAB in comparison to the SDS bilayer.

## 4 Conclusions

This work provides atomistic insights into the interaction between ciprofloxacin and azithromycin antibiotics with the SDS/CTAB bilayer using molecular dynamics simulations. Ciprofloxacin being a zwitterionic compound exhibits a strong molecular interaction with SDS. Also, ciprofloxacin being a hydrophilic drug interacts with the hydrophilic head of the surfactant. On the contrary, the hydrophobic azithromycin interacts with the hydrophobic tail of the surfactant. Our results show that the ciprofloxacin can be separated more effectively in the presence of SDS than CTAB. This conclusion is drawn based on the density profiles along the bilayers, rotational angle of surfactants, and dominant hydrogen bond interaction between SDS and ciprofloxacin. The anionic surfactant SDS is observed to have a better influence on the adsorption of ciprofloxacin than CTAB, whereas the cationic surfactant CTAB exhibits a more pronounced effect on the adsorption of hydrophobic azithromycin compared to SDS. Furthermore, it is evident from our study that the presence of the drugs alters the SDS/CTAB surfactant tilt angle and rotational angle in comparison to pure SDS/CTAB bilayer. A pronounced localization of the ciprofloxacin drug is observed within the SDS bilayers based on the density profiles along the *z*-axis. To summarize, our approach can be generalized to understand the molecular interaction of emerging pharmaceutical pollutants with surfactants.

## Supporting Information Available

Diffusion rates of ciprofloxacin and azithromycin along the *z*-direction of the bilayer (PDF)
