# Supplementary material for: Ciprofloxacin and Azithromycin Antibiotics Interactions with Bilayer Ionic Surfactants: A Molecular Dynamics Study
Source: ACS Omega. 2024 Jul 17;9(30):33174–82. doi: 10.1021/acsomega.4c04673 (PMC11292829; doi:10.1021/acsomega.4c04673)
Supplement: Supplementary file 1 — ao4c04673_si_001.pdf [file ao4c04673_si_001.pdf]

Supplementary Information

Ciprofloxacin and Azithromycin Antibiotics Interactions with Bilayer Ionic Surfactants: A Molecular Dynamics Study

Sriprasad Acharya,[†] Jitendra Carpenter,[†] Muddu Madakyaru,[†] Poulumi Dey,*[,‡]
Anoop Kishore Vatti,*[,†] and Tamal Banerjee,*[,¶]

[†] Department of Chemical Engineering, Manipal Institute of Technology (MIT), Manipal Academy of Higher Education (MAHE), Manipal 576104, Karnataka, India
[‡] Department of Materials Science and Engineering, Faculty of Mechanical, Maritime and Materials Engineering (3mE), Delft University of Technology, 2628 CD Delft, The Netherlands
[¶] Department of Chemical Engineering, Indian Institute of Technology Guwahati, Assam 781039, India

E-mail: P.Dey@tudelft.nl ; anoop.vatti@manipal.edu ; tamalb@iitg.ac.in

To understand the mobility of the drug, diffusion rate 'D' of the respective drug is calculated along the z-direction of the bilayer using the Einstein relation. Einstein relation utilizes slope of the mean-squared displacement versus time profile to estimate the diffusion rate as shown in Eq. S1:

$$D = \frac{1}{6} \lim_{t \to \infty} \frac{d\langle |z(t) - z(0)|^2 \rangle}{dt} \qquad \text{Eq. S1}$$

The calculated diffusion rates are summarized in Table S1. It can be clearly seen that the mobility of the azithromycin, i.e., 1.425×10^{-10} m^2/s is twice higher than the ciprofloxacin, i.e., 0.786×10^{-10} m^2/s. This can be attributed to the higher number of average H-bonds between SDS-Ciprofloxacin than the SDS-azithromycin. Enhanced H-bonds interactions along with steric effect of the hydrophilic head of the surfactant slow down the mobility of the ciprofloxacin. On the other hand, both the drug molecules diffuse at almost at same rate along the z-axis of the CTAB bilayer, this can be attributed to the steric effects. Overall, the drug diffusion rate is lower in CTAB in comparison to the SDS bilayer.

Table S1: Anisotropic diffusion coefficient of the drug molecules along the *z*-direction of the bilayer is summarized. R^2 of the mean squared displacement versus time fit is shown in parenthesis.

	**Diffusion Coefficient of drug (m^2/s)**	
	**Ciprofloxacin**	**Azithromycin**
**SDS Bilayer**	0.786×10^{-10} (0.993)	1.425×10^{-10} (0.985)
**CTAB Bilayer**	0.200×10^{-10} (0.997)	0.207×10^{-10} (0.971)
